# Botulinum toxin injections for Japanese patients with urinary incontinence caused by neurogenic detrusor overactivity: Clinical evaluation of onabotulinumtoxinA in a randomized, placebo‐controlled, double‐blind trial with an open‐label extension

**DOI:** 10.1111/iju.14602

**Published:** 2021-06-01

**Authors:** Masashi Honda, Osamu Yokoyama, Ryosuke Takahashi, Tatsuma Matsuda, Takashi Nakayama, Takao Mogi

**Affiliations:** ^1^ Department of Urology Tottori University Faculty of Medicine Yonago Japan; ^2^ Department of Urology Faculty of Medical Science University of Fukui Fukui Japan; ^3^ Department of Urology Spinal Injuries Center Fukuoka Japan; ^4^ Clinical Development (Oncology) Japan Medical and Development GlaxoSmithKline K.K. Tokyo Japan; ^5^ Biostatistics Japan Medical and Development GlaxoSmithKline K.K. Tokyo Japan; ^6^ Clinical Development (Specialty) Japan Medical and Development GlaxoSmithKline K.K. Tokyo Japan

**Keywords:** botulinum toxin type A, neurogenic detrusor overactivity, onabotulinumtoxinA, randomized controlled trial, urinary incontinence

## Abstract

**Objective:**

To assess the efficacy and safety of botulinum toxin treatment (onabotulinumtoxinA 200 units) for Japanese patients with neurogenic detrusor overactivity caused by spinal cord injury or multiple sclerosis.

**Methods:**

Patients with urinary incontinence refractory to pharmacological treatment were enrolled and randomized in a phase III trial. A single dose of onabotulinumtoxinA (*n* = 11) or placebo (*n* = 10) was given in the double‐blind phase, and repeat injections of onabotulinumtoxinA were given in the subsequent open‐label phase. Outcomes included urinary incontinence episodes, urodynamics, patient‐reported outcomes and adverse events.

**Results:**

The onabotulinumtoxinA group showed a numerically greater reduction in the number of urinary incontinence episodes per day than the placebo group, with the difference between the groups at week 6 of −3.02 (95% confidence interval −5.85 to −0.19). The onabotulinumtoxinA group also showed greater improvements in urodynamic assessments. Adverse events related to onabotulinumtoxinA injections were hematuria, urinary retention, urinary bladder hemorrhage, autonomic dysreflexia and epididymitis. Most events were deemed mild or moderate.

**Conclusions:**

Intradetrusor injections of onabotulinumtoxinA are efficacious and tolerable for Japanese patients with neurogenic detrusor overactivity‐related symptoms that are difficult to manage with anticholinergics and/or β_3_‐adrenergic receptor agonists.

**Abbreviations & Acronyms**
AE: adverse event
BoNTA: onabotulinumtoxinA
CI: confidence interval
CIC: clean intermittent catheterization
FAS: full analysis set
IDC: involuntary detrusor contraction
MCC: maximum cystometric capacity
MS: multiple sclerosis
NDO: neurogenic detrusor overactivity
P_maxIDC_
: maximum detrusor pressure during the first involuntary detrusor contraction
PVR: post‐void residual
QOL: quality of life
SCI: spinal cord injury
SD: standard deviation
SE: standard error
TBS: treatment benefit scale
TC: treatment cycle
UI: urinary incontinence

## Introduction

NDO is a urodynamic observation characterized by IDC during the filling phase, and its primary cause is neurological conditions, such as SCI and MS. NDO frequently induces UI, which can negatively impact QOL, and high intravesical pressure, which can damage the upper urinary tract.^1^, ^2^, ^3^ Pharmacological treatments including anticholinergics are generally used for NDO to decrease intravesical pressure and improve continence. However, their use is often limited by insufficient efficacy and/or intolerable side‐effects, such as constipation and dry mouth.^4^, ^5^

Botulinum toxin type A is a neuromuscular blocker that inhibits the presynaptic release of acetylcholine at the parasympathetic nerve terminal, and causes the detrusor muscle to relax.^6^ At present, BoNTA is the only botulinum toxin formulation that has been approved worldwide for the treatment of NDO. It has been shown to improve NDO in several randomized trials carried out mainly in Europe and North America.^7^, ^8^, ^9^ Its use is recommended worldwide as a treatment option for NDO patients who cannot have satisfactory results with prior therapies (e.g. anticholinergics).^10^, ^11^

In Japan, before carrying out the present trial, BoNTA was assessed only in an open‐label study with a relatively small number of NDO patients and its use had not been approved for NDO.^12^ Thus, the present trial was carried out to evaluate its efficacy and safety in Japanese NDO patients who had UI refractory to other drugs in support of registration in Japan.

## Methods

### Participants

Patients aged 20 years or older with NDO secondary to SCI (with neurological injury level at C5 or below) or MS were enrolled. They had to have six or more episodes of UI recorded in a 3‐day diary, with no more than one UI‐free day. The patients were all refractory to anticholinergics and/or β_3_‐adrenergic receptor agonists, experiencing ineffectiveness or intolerability. These NDO medications were maintained on the same treatment regimen, if necessary, throughout the double‐blind phase (TC 1), but could be reduced or discontinued in the open‐label phase (TCs 2 and 3). Patients could join regardless of CIC use; however, they had to provide consent to use CIC when the investigator deemed it necessary. The exclusion criteria included any diseases, functional abnormalities or bladder surgery that might have affected bladder functions; previous treatment of any urological condition with botulinum toxin; and a PVR urine volume of >200 mL in patients with spontaneous voiding. Eligible patients were randomly allocated (at a ratio of 1:1) to BoNTA 200 U or placebo, while being stratified according to NDO etiology (SCI with neurological injury level at C5 to C8, T1 or below, or MS).

### Design

**Fig. 1:**
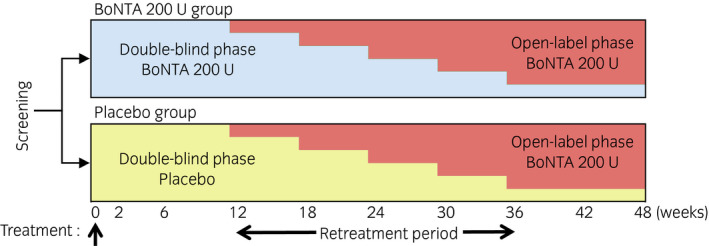
Study design.

**Fig. 2:**
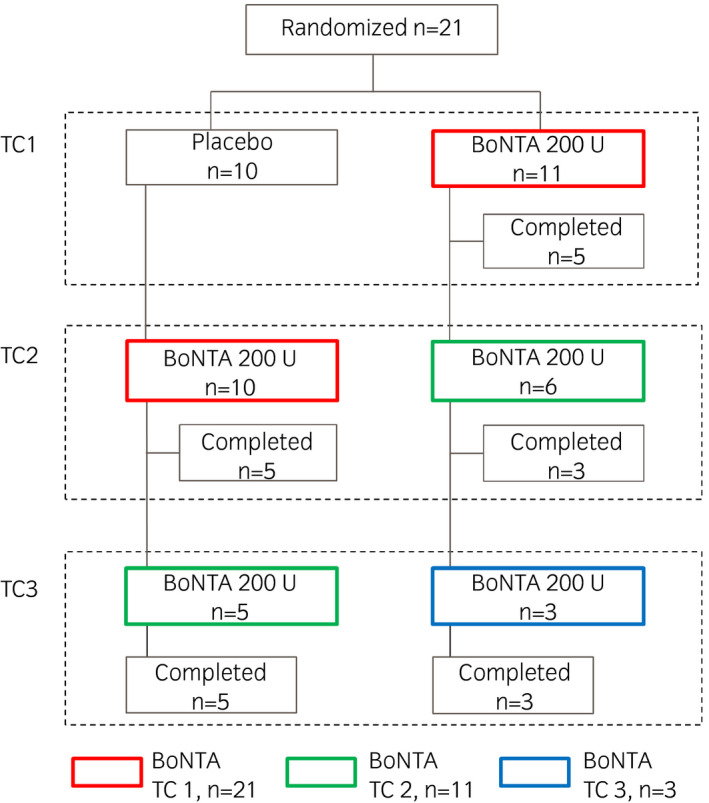
Patient flow through trial.

This phase III trial included a randomized, placebo‐controlled, double‐blind phase followed by an open‐label retreatment phase (ClinicalTrials.gov NCT02849418). The study was carried out in Japan from October 2016 to December 2018, with approval from the institutional review board obtained for all 12 study sites and in accordance with Good Clinical Practice regulations. The trial comprised a screening period (up to 4 weeks before the first dose) and a treatment period (48 weeks) consisting of two phases: (i) a comparison was made between single injections of BoNTA 200 U and placebo in the double‐blind phase; and (ii) repeat injections of BoNTA 200 U were given in the subsequent open‐label phase (Fig. 1). BoNTA TC 1 (*n* = 21; red cells in Fig. 2) was defined as the first TC with BoNTA, including patients who were treated with BoNTA in the double‐blind phase, as well as patients who were treated with placebo in the double‐blind phase and then received BoNTA in the subsequent cycle. Similarly, BoNTA TC 2 (*n* = 11; green cells in Fig. 2) and BoNTA TC 3 (*n* = 3; blue cell in Fig. 2) were defined as the second and third TCs with BoNTA, respectively.

### Treatment

Patients were given prophylactic antibiotics before and after treatment, and on the day of treatment. Local anesthesia could be intravesically instilled (with or without sedation) at the discretion of the investigator. General anesthesia was used for SCI patients with neurological injury levels of C5 to C8, according to local site practice due to the potential for developing autonomic dysreflexia from the injection procedure. A rigid or flexible cystoscope with a 22‐G needle attached (BoNee; Coloplast, Humlebaek, Denmark) was used to inject 200 U of BoNTA (BOTOX; Allergan, Dublin, Ireland) and placebo, both reconstituted with 30 mL of saline. Each dose was equally divided into 30 sites in the detrusor muscle, sparing the dome and trigone. Patients could request retreatment of BoNTA 200 U at 12 weeks or later, up to 36 weeks after the first treatment, and then they entered the open‐label phase, if qualified, to receive retreatment of BoNTA 200 U, as shown in Figure 1. To be qualified for retreatment, patients had to have experienced at least four episodes of UI, with no more than one urinary incontinence‐free day, recorded in the bladder diary during the three consecutive days of the week before the qualification for retreatment visit.

### Evaluations of efficacy and safety

A 3‐day diary was utilized to gather data during the screening period and in the week before each post‐treatment visit. The primary end‐point was the change from baseline in the number of daily UI episodes at week 6 after a single dose. Secondary end‐points included the changes from baseline in MCC and P_maxIDC_ after a single dose, the change from baseline in the number of daily UI episodes after repeated doses at week 6 for each total number of BoNTA treatment and time to patient request for retreatment. Patient‐reported outcome after a single dose and in the BoNTA TCs 2 and 3 was assessed using TBS.^13^ The patients with a response of “greatly improved” or “improved” among response options (greatly improved, improved, not changed or worsened) were considered to have a positive response on the TBS.

With respect to safety, AEs were collected, PVR urine volume was measured and CIC use for urinary retention or elevated PVR was recorded. Clinical laboratory tests (i.e. chemistry, hematology and urinalysis including urine culture) were carried out, as well as monitoring of vital signs, ultrasonographic observation of the bladder and kidney and 12‐lead electrocardiogram recording. Blood samples were collected to assess immunogenicity at screening and at the end of the study.

### Statistical analysis

The primary analyses were carried out for the FAS (all randomized patients with one or more post‐baseline assessments of efficacy). Safety analyses were carried out for the safety population (patients who had received at least one dose of any study drug). A sample size was determined as 30 patients based on enrollment feasibility. The study provided a 26% power to detect treatment differences for the change from baseline in the mean number of daily UI episodes at a significance level of 5%, with the assumption that the mean difference would be −1.5 episodes per day, and its SD would be 3.0 episodes per day. For the analysis of the primary end‐point, a mixed model for repeated measures was used with treatment, visit, baseline value, treatment‐by‐visit interaction and baseline value‐by‐visit interaction as fixed effects. The present study was not designed to evaluate formal statistical hypotheses. Therefore, the multiplicity was not adjusted for any end‐points, and sensitivity/supportive analyses were not planned. Considering the small target number of patients, no subgroup analyses were planned.

## Results

Although 30 patients were to be randomized according to the initial plan, recruitment was closed when 21 patients (11 patients in the BoNTA group, 10 patients in the placebo group) were randomized into the study and received study treatment in TC 1 because of difficulty in enrollment of patients. All 21 patients had SCI; one patient with MS was screened, but did not fulfill the eligibility criteria.

The 10 patients in the placebo group all entered the open‐label phase and received retreatment with BoNTA 200 U; in contrast, approximately half the patients (5/11) in the BoNTA group completed the 48‐week study period without receiving retreatment of BoNTA 200 U. In total, BoNTA 200 U was given more than once to 11 patients, three of whom received it three times (Fig. 2). No patients discontinued the study.

The baseline demographics and disease characteristics of patients are shown in Table 1. A lack of efficacy was the primary reason for being inadequately managed with prior NDO medications in all patients.

**Table 1:** Baseline demographics and disease characteristics (FAS population)

	Placebo (*n* = 10)	BoNTA 200 U (*n* = 11)
Age (years)	47.2 ± 18.29	50.9 ± 14.12
Male, *n* (%)	9 (90%)	8 (73%)
Duration of NDO (years)	7.56 ± 7.058	16.35 ± 13.314
Etiology of NDO, *n* (%)		
SCI (C5 to C8)	0	1 (9%)
SCI (≤T1)	10 (100%)	10 (91%)
MS	0	0
American Spinal Injury Association impairment scale of SCI patients, *n* (%)		
A: Complete	7 (70%)	8 (73%)
B: Sensory incomplete	0	1 (9%)
C: Motor incomplete	0	0
D: Motor incomplete	2 (20%)	2 (18%)
E: Normal	1 (10%)	0
Void pattern, *n* (%)		
CIC only	5 (50%)	5 (45%)
Mixed (spontaneous void and CIC)	1 (10%)	3 (27%)
Spontaneous void only	1 (10%)	1 (9%)
CIC and intermittent balloon catheter	3 (30%)	2 (18%)
Existing medication use, *n* (%)		
Anticholinergics only	8 (80%)	9 (82%)
β_3_‐adrenergic receptor agonist only	1 (10%)	0
Anticholinergics and β_3_‐adrenergic receptor agonist	1 (10%)	2 (18%)
Primary reasons for being inadequately managed with prior NDO medication, *n* (%)		
Lack of efficacy	10 (100%)	11 (100%)
Adverse event	0	0
No. daily UI episodes	5.17 ± 2.626	3.91 ± 2.034
MCC (mL)	241.15 ± 121.514	251.64 ± 131.759
P_maxIDC_ (cmH_2_O)	51.51 ± 28.595	44.91 ± 31.772

All data are expressed as the mean ± SD other than the data expressed as *n* (%).

### Efficacy of single‐dose treatment (TC 1: double‐blind phase)

**Fig. 3:**
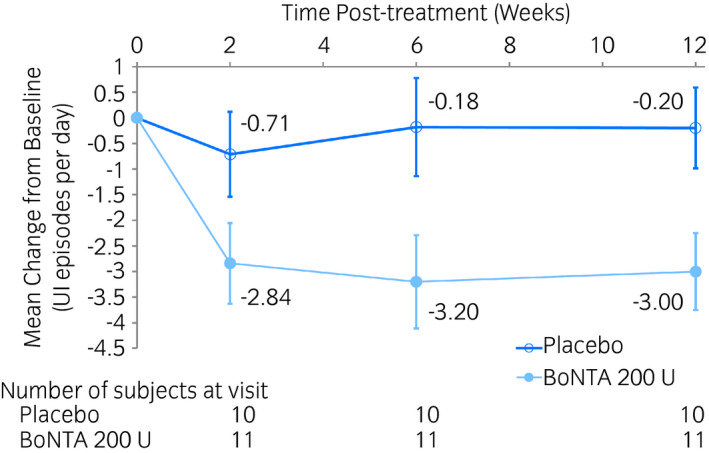
Change from baseline in the mean number of daily UI episodes for treatment cycle 1. Values: adjusted mean; error bars: SE. The data were analyzed using a mixed model for repeated measures with treatment, visit, treatment‐by‐visit interaction, baseline value and baseline value‐by‐visit interaction as fixed effects.

The change from baseline in the mean number of daily UI episodes at week 6 was −3.20 in the BoNTA group and −0.18 in the placebo group (Fig. 3, Table 2). The difference between the treatment groups was −3.02 (95% CI −5.85 to −0.19), with a numerically greater reduction noted in the BoNTA group (Table 2).

**Table 2:** Change from baseline in UI episodes and urodynamic parameters to post‐treatment week 12 of treatment cycle 1 (FAS population)

	Placebo (*n* = 10)	BoNTA 200 U (*n* = 11)	Difference *vs* placebo (95% CI)
No. daily UI episodes†			
Week 2	−0.71 ± 0.829	−2.84 ± 0.789	−2.14 (−4.59, −0.31)
Week 6	−0.18 ± 0.957	−3.20 ± 0.911	−3.02 (−5.85, −0.19)
Week 12	−0.20 ± 0.790	−3.00 ± 0.752	−2.81 (−5.14, −0.47)
MCC (mL)			
Week 6	59.32 ± 39.669	159.57 ± 37.821	100.25 (−14.96, 215.45)
P_maxIDC_ (cmH_2_O)			
Week 6	−0.166 ± 4.5195	−20.785 ± 5.2187	−20.619 (−35.814, −5.424)
No. patients with no IDC, *n* (%)			
Week 6	2 (20)	5 (45)	–

All data except for “Number of patients with no IDC, *n* (%)” are expressed as adjusted mean ± SE.

†Mixed model for repeated measures with treatment, visit, treatment‐by‐visit interaction, baseline value and baseline value‐by‐visit interaction as fixed effects.

Compared with the placebo group, patients in the BoNTA group had an increase in MCC and a decrease in P_maxIDC_ at week 6 (Table 2). The proportion of patients without any IDC at week 6 was higher in the BoNTA group (45%) as compared with the placebo group (20%; Table 2).

**Fig. 4:**
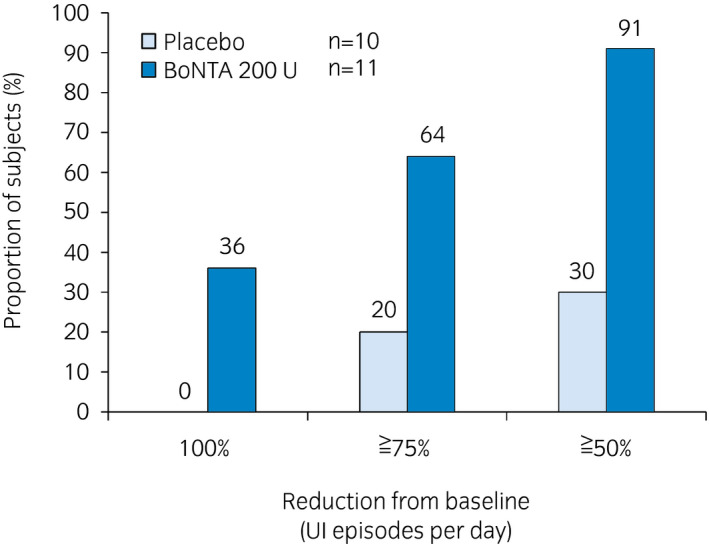
Proportion of patients attaining 100%, ≥75% and ≥50% reduction from baseline in the number of daily UI episodes at week 6 of treatment cycle 1.

In addition, the proportion of patients attaining 100% reduction in daily UI episodes from baseline (i.e. “dry”) at week 6 was higher in the BoNTA group (36%) as compared with the placebo group (0%; Fig. 4). The BoNTA group also included a higher proportion of patients with a positive response on the TBS at week 6 (Table 3).

**Table 3:** Number and proportion of patients with a positive treatment response on the treatment benefit scale up to post‐treatment week 12 of treatment cycle 1 (FAS population)

	Placebo *n* = 10	BoNTA 200 U *n* = 11	Odds ratio (95% CI)
*n* (%)†			
Week 2	1 (10)	6 (55)	10.80 (1.00, 117.00)
Week 6	2 (20)	10 (91)	40.00 (3.05, 524.83)
Week 12	3 (30)	8 (73)	6.22 (0.94, 41.38)

†A positive treatment response was defined as a score of either 1 (greatly improved) or 2 (improved). Missing data due to any reason were regarded as non‐response until week 12 after first treatment. Statistics were to be missing when the number of responders was too low/high to estimate the value.

### Efficacy of repeated‐dose treatment

**Fig. 5:**
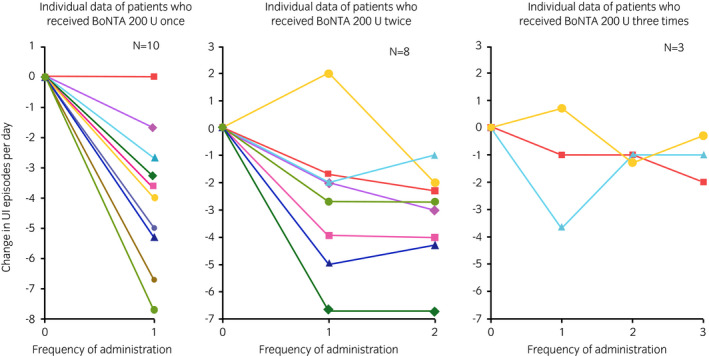
Change from baseline in the mean number of daily UI episodes at week 6 for each total number of BoNTA treatments.

The change from baseline in the mean number of daily UI episodes at week 6 for each total number of BoNTA treatment is shown in Figure 5. The mean number of daily UI episodes at week 6 was reduced from the study baseline for most patients receiving repeated doses. In the majority of patients, the changes from the study baseline in the mean number of daily UI episodes at week 6 were similar among the BoNTA TCs. The proportions of patients attaining 100% reduction from baseline in daily UI episodes (“dry”) at week 6 of BoNTA TCs 2 and 3 were 36% (4/11) and 33% (1/3), respectively. The proportions of patients with a positive response on the TBS at week 6 of BoNTA TCs 2 and 3 were 82% (9/11) and 67% (2/3), respectively.

### Duration of efficacy

**Fig. 6:**
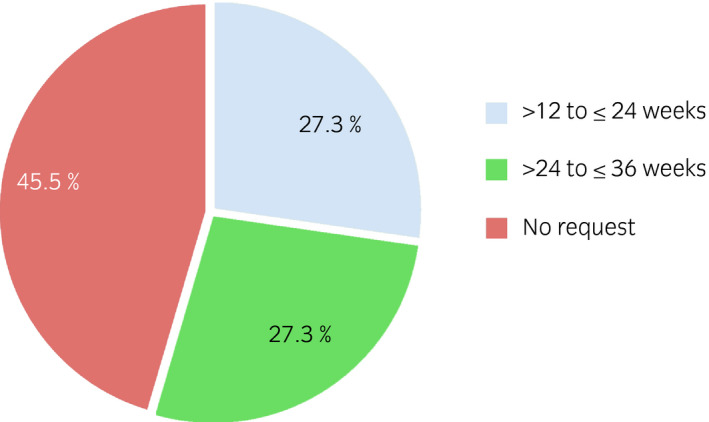
Time from first injection to the patients’ first request for retreatment in the BoNTA group.

In the BoNTA group, 54.5% (6/11) requested retreatment with BoNTA within 36 weeks after the first treatment and 45.5% (5/11) did not request retreatment (Fig. 6). The median time to patient request for retreatment was 246.0 days in the BoNTA group and 84.5 days in the placebo group. The median time to qualification for retreatment was 246.0 days in the BoNTA group and 85.0 days in the placebo group.

### Safety

The incidence of AEs during the first 12 weeks of TC 1 was 73% (8/11) in the BoNTA group and 50% (5/10) in the placebo group (Table 4). Treatment‐related AEs in the BoNTA group were hematuria, urinary retention and autonomic dysreflexia (9% [1/11] each), which were localized to the urinary tract except for autonomic dysreflexia. All AEs reported in the BoNTA group were deemed mild or moderate.

**Table 4:** Summary of all AEs in the first 12 weeks of treatment cycle 1 (safety population)

AE	Placebo, *n* = 10 *n* (%)	BoNTA 200 U, *n* = 11 *n* (%)
All AEs	5 (50)	8 (73)
Decubitus ulcer	2 (20)	1 (9)
Hematuria	1 (10)	1 (9)
Fall	1 (10)	1 (9)
Urinary tract infection†	0	2 (18)
Appendicitis	0	1 (9)
Nasopharyngitis	0	1 (9)
Abdominal pain lower	0	1 (9)
Urinary retention‡	0	1 (9)
Autonomic dysreflexia	0	1 (9)
Ligament sprain	0	1 (9)
Back pain	0	1 (9)
Muscular weakness	0	1 (9)
Cardiac failure	0	1 (9)
Pyrexia	0	1 (9)
Dizziness	0	1 (9)
Headache	0	1 (9)
Nail disorder	1 (10)	0
Urticaria	1 (10)	0
Ileus	1 (10)	0
Tooth loss	1 (10)	0
Skin abrasion	1 (10)	0
Sleep apnoea syndrome	1 (10)	0

†Urinary tract infections were reported as AEs regardless of the presence of symptoms when the urinalysis result was positive (bacteriuria with ≥10^5^ colony forming units/mL and leukocyturia with >5/high power field).

‡The definition of urinary retention was when a patient who had an increased PVR urine volume required CIC; PVR urine volume of ≥350 mL (regardless of symptoms) or PVR urine volume ≥200 mL and <350 mL, and reported associated symptoms (e.g. voiding difficulties or sensation of bladder fullness) for which the investigator deemed CIC to be necessary.

Adverse events reported for two or more patients in BoNTA TCs 1‐–3 are shown in Table 5. Urinary tract infection was the most common AE, and its incidence in BoNTA TCs 1–3 was 24%, 27% and 67%, respectively. Treatment‐related AEs reported in any BoNTA TCs were hematuria (14%, 3/21), autonomic dysreflexia (10%, 2/21), urinary retention (5%, 1/21), urinary bladder hemorrhage (5%, 1/21) and epididymitis (5%, 1/21), which were also localized to the urinary tract, except for autonomic dysreflexia and epididymitis. One patient with spontaneous voiding in the BoNTA group used CIC during the study period, as this patient's PVR urine volume at week 2 of TC 1 was 457.3 mL, showing an increase of 382.3 mL from baseline. In this patient, the PVR urine volume dropped below 200 mL at week 36.

**Table 5:** Summary of AEs reported for two or more patients in BoNTA treatment cycles (safety population)

AE	BoNTA TC 1 *n* = 21 *n* (%)	BoNTA TC 2 *n* = 11 *n* (%)	BoNTA TC 3 *n* = 3 *n* (%)	Overall, *n* = 21 *n* (%)
All AEs	14 (67)	9 (82)	3 (100)	18 (86)
Urinary tract infection†	5 (24)	3 (27)	2 (67)	7 (33)
Decubitus ulcer	2 (10)	2 (18)	0	4 (19)
Pyrexia	2 (10)	1 (9)	1 (33)	4 (19)
Nasopharyngitis	2 (10)	1 (9)	0	3 (14)
Haematuria	2 (10)	0	1 (33)	3 (14)
Eczema	2 (10)	0	0	2 (10)
Autonomic dysreflexia	2 (10)	1 (9)	0	2 (10)
Headache	1 (5)	1 (9)	0	2 (10)

†Urinary tract infections were reported as AEs regardless of the presence of symptoms when the urinalysis result was positive (bacteriuria with ≥10^5^ colony forming units/mL and leukocyturia at >5/high power field).

Three events of autonomic dysreflexia occurred in two patients. The events were considered related to the injection procedure, as they occurred on the day of study treatment or the following day. The patients’ neurological injury levels were T1 or below, and neither patient received general anesthesia during treatment administration. Most AEs reported during the study period were mild or moderate in severity. No death or treatment discontinuation was reported. No significant changes or findings were detected by clinical laboratory tests, monitoring of vital signs, ultrasonographic observation of the bladder and kidney or 12‐lead electrocardiogram recording, although urinalysis results showed between‐group differences associated with the urinary tract infections reported. No seroconversions for neutralizing antibodies against BoNTA were reported.

## Discussion

In the treatment of NDO, primary goals are to protect the upper urinary tract by lowering intravesical pressure and increasing bladder capacity, and improve QOL by reducing incontinence.^14^, ^15^ These parameters were improved by treatment with BoNTA 200 U in the present study. At week 6 after the first dose, the BoNTA group showed numerically greater improvements than the placebo group in the primary and secondary end‐points associated with NDO symptoms. These results were in line with those observed in the randomized trials carried out mainly in Europe and North America.^7^, ^8^, ^9^ The proportion of patients with a positive response on the TBS at week 6 was higher in the BoNTA group as compared with the placebo group. Because TBS is an instrument used to measure overall patient perceived outcome after treatment, the positive response on the TBS suggests that the QOL of patients was improved. In addition, repeated treatment with BoNTA showed a tendency toward improvements in the UI reduction and TBS, although the number of patients was limited. From a safety perspective, BoNTA 200 U was tolerated well, with AEs localized primarily to the urinary tract in both the double‐blind and open‐label phases. These results closely resemble those obtained in the randomized trials carried out mainly in Europe and North America, which did not enroll patients with SCI and neurological injury level of C5 to C8.^7^, ^8^, ^9^ Because the present study allowed enrolment of those patients, general anesthesia had to be used in patients with SCI in thoracic spinal cord 6 or above, as these patients were considered to have a relatively high risk of autonomic dysreflexia.^16^, ^17^, ^18^ Actually, general anesthesia was used to prevent autonomic dysreflexia in one patient with a neurological injury level of C6 in the BoNTA group (TC 1) and three patients with neurological injury level of T1 or below in the placebo group (one patient in TC 1, and two patients in TCs 1 and 2). In these patients, autonomic dysreflexia did not occur during the injection procedure, although three events of autonomic dysreflexia were reported in the patients with neurological injury level of T1 or below who did not receive general anesthesia. These findings suggest that general anesthesia was effective in reducing the risk of autonomic dysreflexia. In addition, spinal anesthesia might also be used to prevent autonomic dysreflexia,^19^ and future investigations on its usefulness are warranted.

One limitation of the present study was that careful interpretation was required because of the small sample size due to difficulties with recruitment. In addition, no patients with MS were analyzed. However, given that BoNTA improved symptoms and urodynamic parameters in patients with SCI and MS in the trials carried out mainly in Europe and North America,^20^ and that ethnic differences are unlikely to occur in the response to BoNTA, the favorable results with SCI patients in the present study suggest that BoNTA might be effective in Japanese patients with MS as well.

In conclusion, intradetrusor injections of BoNTA 200 U reduced the number of UI episodes and improved urodynamic parameters in Japanese NDO patients who had UI refractory to anticholinergics and/or β_3_‐adrenergic receptor agonists. The treatment was well tolerated, and most AEs were mild or moderate. The results of this trial suggest that BoNTA might be a useful option for Japanese NDO patients who cannot be managed adequately with other drugs due to ineffectiveness and/or intolerability.

## Conflict of interest

Masashi Honda has received consultancy fees from GlaxoSmithKline K.K. Osamu Yokoyama has received consultancy fees from GlaxoSmithKline K.K, Astellas, Kissei, Kyorin, Nippon Shinyaku, Pfizer and Taiho, and grants from Astellas, Eisai, Kissei, Taiho and Takeda. Takao Mogi, Tatsuma Matsuda and Takashi Nakayama are employees of GlaxoSmithKline K.K. Ryosuke Takahashi has no conflict of interest to declare.

## Data Availability

Anonymized individual participant data and study documents can be requested for further research from www.clinicalstudydatarequest.com.

## Appendix

Principal investigators and their affiliations: Takeya Kitta, Hokkaido University Hospital; Takahiro Yoneyama, Hirosaki University School of Medicine and Hospital; Takashige Namima, Tohoku Rosai Hospital; Tomonori Yamanishi, Dokkyo Medical University Hospital; Yosuke Matsuta, University of Fukui Hospital; Norifumi Sawada, University of Yamanashi Hospital; Atsushi Sengoku, Hyogo Rehabilitation Center Hospital; Atsushi Takenaka, Tottori University Hospital; Toyohiko Watanabe, Okayama University Hospital; Yasuyo Yamamoto, Tokushima University Hospital; Ryosuke Takahashi, Spinal Injuries Center; Hideki Sakai, Nagasaki University Hospital.
